# Customers’ Experiences of Compassion and Brand Attitude: Evidence From Low-Cost Carriers

**DOI:** 10.3389/fpsyg.2021.687155

**Published:** 2021-07-08

**Authors:** Sung-Hoon Ko, Yongjun Choi, Jongsung Kim

**Affiliations:** ^1^Graduate School of Education, Kyonggi University, Suwon, South Korea; ^2^College of Business Administration, Hongik University, Seoul, South Korea; ^3^College of Liberal Arts and Interdisciplinary Studies, Kyonggi University, Suwon, South Korea

**Keywords:** compassion, positive emotion, brand image, brand attitude, airlines, low-cost carriers

## Abstract

The purpose of this study is to examine the effect of compassion experienced by low-cost carriers customers on their brand attitudes. Specifically, this study aims to unbox the mechanisms through how the customers’ experiences of compassion influence the formation of positive brand attitudes. Using the data from 423 low-cost carriers customers in South Korea, this study found that the more low-cost carriers’ customers experience compassion, the more positive their brand attitudes are toward the low-cost carriers. Notably, this study demonstrated that the positive relationship between compassion and brand attitudes is serially mediated by positive emotion and positive brand image. The results from this study contribute to the literature on the airline industry by examining the roles of compassion, which is relatively new to the field, and also provide practical insights for a low-cost carrier to come up with competitive strategies to achieve a competitive advantage over its competitors in the industry.

## Introduction

The continued growth and success of newly entered low-cost carriers (e.g., Southwest Airlines in the United States; Ryan Air and Easy Jet in Europe; and Air Asia, Nok Airlines, One-two-go Airlines, Tiger Airways, and Value Air in Asia) have accelerated more new competitors to enter the market (Baker and Mc, 2013). The first low-cost carrier, British South America Airways, started in 1946 with a test flight from London Heathrow Airport to Buenos Aires in South America. Southwest Airlines began operations in 1967, and have been operating alongside full-service carriers in the U.S. Ryan Air also started operations in 1986, and have been carrying the largest number of international passengers worldwide (Baker and Mc, 2014). Additionally, Malaysia’s Air Asia has been operating in Southeast Asia, with sales exceeding 1 trillion since the 9/11 terrorist attack in 2001. Therefore, the number of customers using low-cost carriers has increased exponentially; accordingly, it is more important for low-cost carriers to increase their customers’ brand loyalty.

South Korea reformed the air transport business licensing and eased airline establishment licensing to promote new airlines’ entry into the market and secure international competitiveness. The range of airlines was relatively limited from customers’ perspective in South Korea until the advent of low-cost carriers; all airlines provided relatively similar services and fares, as such service quality or brand image did not act as an important factor in the customer’s choice of airline. However, Hansung Airlines, the predecessor of T’way Air, launched its first service as a low-cost carrier in 2005 and launched international flights in 2009. Six low-cost carriers operate in South Korea as of 2018. According to an announcement by the Ministry of Land, Infrastructure, and Transport in February 2018, the number of international passengers who used domestic low-cost carriers was 20,302,100 in 2017, an increase of 41.9% from the previous year. This advancement of low-cost carriers can be attributed to low prices amid the economic recession and increased overseas travel among young people.

Although low fares are still the strongest predictor of one’s intention to use low-cost carriers over full-service carriers, customers’ perceptions of service quality also matter in such decisions (Pan and Truong, 2018). Supporting that, Pearson et al. (2015) showed that, for both low-cost and full-service carriers, service reputation is one of the core intangible resources to gain competitive advantages. That is, even though the major differences between low-cost carriers and full-service carriers are price and service quality (Dobruszkes, 2006; Han et al., 2014), customers still expect high levels of service quality from the low-cost carriers. Likewise, despite the remarkable advancement, low-cost carriers that simply offer low fares cannot survive fierce competition with full-service carriers in Korea. According to a recent survey by a Korean market research company (Embrain Trend Monitor), customer satisfaction with low-cost carriers, including even the low-cost carriers owned by Korean full-service carriers, did not reach the level of satisfaction with full-service carriers. That is, low-cost carriers did not exceed the brand power of full-service carriers. Therefore, they need to reinforce their brand power as a real “airline” beyond the mere image of a low-cost airline. Especially, given the importance of high-quality in-flight services for Korean travelers, improving high-quality services for Korean low-cost carriers are vital for Korean low-cost carriers to have more travelers return to their flight services (Han et al., 2019). In addition, given the dearth of empirical studies on low-cost carriers in Korea (Khan et al., 2019), Korean low-cost airlines are ideal to explore the roles of customers’ experiences of compassion.

Research related to airline service has mainly focused on service quality and customer satisfaction attributes of general airlines. For example, studies have analyzed the relationship between brand personality and satisfaction with the service quality of low-cost carriers (Wongleedee, 2017), brand image and the barriers to customer satisfaction (Koklic et al., 2017), and service quality on performance variables (Curras-Perez and Sanchez-Garcia, 2016). However, albeit airline customers interact with airlines or airline employees in various contexts, from booking through boarding to flying, affecting their formation of either positive or negative perceptions toward the airlines, the previous studies are somewhat limited in that they did not explicitly capture the interpersonal processes between customers and airlines. Especially since air travel can entail many interpersonal situations, while using the airlines services, customers often experience suffering (Bejou and Palmer, 1998; Heller, 2003; Bor, 2007) that are likely to negatively affect their attitudes toward the carriers. Thus, the extent to which low-cost carriers customers experience compassion, “an interpersonal process involving the noticing, feeling, sense-making, and acting that alleviates the suffering of another person” (Dutton et al., 2014, p. 278), can serve important roles in the customers’ formation of either positive or negative perceptions toward the low-cost carriers.

Therefore, this study aims to empirically investigate the effect of compassion experienced by customers on their brand attitude toward low-cost carriers. Particularly, it grounded on the psychology, marketing, and management literatures. This study examines the double mediation effect by focusing on customers’ positive emotion and brand image through which compassion experienced by their customers influences the formation of their brand attitude. That is, this study intends to demonstrate that customers who experience compassion while using low-cost airlines are likely to experience more positive emotions which, in turn, helps the formation of positive brand images and attitudes. Accordingly, given the dearth of research on the customers’ experiences of compassion, this study provides theoretical implications to the existing literature by examining customers’ experience of compassion and its effects that have received relatively less attention in prior research on airlines services and consumer behaviors. Practically, this study also provides implications for low-cost carriers about how to improve low-cost carriers’ service level and spreading an empathetic culture among employees and crew critical for gaining competitive advantages in the airlines industry.

## Theoretical Background and Hypotheses Development

**Figure 1:**
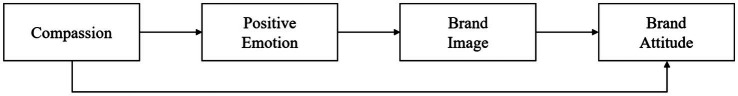
Conceptual model.

Our conceptual model is presented in Figure 1.

Consumer compassion is a dynamic process in which emotional reactions are projected into the customer’s consciousness, particularly the consumer’s imagination about others’ experiences (Meyer et al., 2019). Specifically, the consumer’s empathy consists of cognitive and emotional empathy. Whereas cognitive empathy refers to the degree of understanding and sympathizing toward objects, emotional empathy means not only understanding but reaching a state of deep emotional immersion as if you experienced the other’s situation (Ephron, 2006). Particularly, consumers’ empathetic reactions contribute to creating a relationship by projecting themselves into the brand, thereby eliciting a favorable response.

We expect that consumers’ experiences of compassion while using low-cost carriers could help them form positive brand attitudes toward the related low-cost carriers. Brand attitude is a consumer’s emotional state that evaluates positive or negative reactions to a corporate brand. Specifically, Fishbein (1963) defines brand attitude as the overall psychological state of an individual to a brand. Brand attitude is developed by forming an attachment to the brand’s attributes and activities in a specific situation. This is important because it is a criterion for evaluating the brand as a whole regarding the actions and choices of consumers (Faircloth et al., 2001). In addition, brand attitudes are formed and can be changed through the modification of one’s cognitive structure (Lutz, 1975) and experiences (He et al., 2016). Previous studies on service quality also provide support that service quality customers perceive affect the levels of brand associations (Alexandris et al., 2008), brand loyalty (Hemsley-Brown and Alnawas, 2016), and behavioral intentions (Carlson and O’Cass, 2010).

Likewise, the extent to which customers experience compassion while using low-cost carriers services can capture their perceptions of low-cost carriers service quality. Specifically, customers using low-cost carriers will focus on airlines’ compassionate services cognitively and experience them emotionally, reaching emotional empathy and thereby evaluating low-cost carrier brands more favorably (Wuthnow, 2012; Childs and Kim, 2019) because they are likely to perceive that they are receiving genuine cares. As such, when customers experience spiritual, material, and temporal compassion, they will develop a positive brand attitude overall, such as liking and trusting the brand.

*H1*: Low-cost carriers’ customers’ experiences of compassion are positively related to brand attitudes.

Lilius et al. (2008) define compassion as an emotion that responds to and sympathizes with others’ suffering and argue that individuals who experience compassion forms positive emotions, such as pride, joy, inspiration, and comfort. Likewise, although the majority of research was conducted in the organizational setting using the employees sample, positive organizational psychologists also support that the experiences of compassion lead to positive emotional responses (Kahn, 1993, 1998; Hallowell, 1999; Folkman and Moskowitz, 2000; Dutton, 2003; Frost, 2003; Dutton et al., 2010). In this study’s context, according to the affective events theory (Weiss and Cropanzano, 1996), when customers experience compassion while using the low-cost carrier services (e.g., booking, rescheduling) or during interactions with employees or flight crews, they are likely to interpret them as positive events, invoking positive emotions. For example, when low-cost carrier employees or crew express compassion toward customers’ problems or requests, customers are likely to perceive that they are genuinely cared for, leading to positive emotions. Previous studies on service quality also provide the support that customers perceiving high levels of service quality are likely to have more positive affective responses to the services (Baumann et al., 2007; Gracia et al., 2011). Similarly, the positive emotion appears high when customers are satisfied with a product or service (Wirtz et al., 2000). Therefore, we predict the following:

*H2*: Low-cost carriers’ customers’ experiences of compassion are positively related to the positive emotions.

According to the pleasure, arousal, and dominance model developed by Mehrabian and Russell (1974), an individual’s emotional reactions (i.e., pleasure, arousal, and dominance) are manifested by the environment, which acts as an important variable in determining attitudes and behaviors. Accordingly, Fredrickson (2001) argues that people’s thoughts, actions, and relationships expand when they experience positive emotions. Thus, when individuals experience positive emotions, they are more likely to recall positive memories, interpret related situations or objects more positively, and be connected to external expressions like extending kind behaviors toward others (Sallam, 2014). Similarly, the broaden-and-build model of positive emotion (Fredrickson, 1998, 2001) shows that positive emotion caused by a specific event arouses an individuals’ attention, having them recognize the experienced event anew, build confidence, and alleviate negative emotions effectively. Therefore, the experiences of compassion while using low-cost carriers are likely to allow the customers to experience positive emotions toward a specific low-cost carrier which, in turn, have them feel persistent emotional attachment (Oliver, 1980; Dick and Basu, 1994) and build positive brand images because they can easily retrieve positive experiences associated with the low-cost carriers they used. In sum, we predict that customers who report positive emotions owing to their experiences are likely to form emotional attachments helping them form positive images toward the related low-cost carrier.

*H3*: Low-cost carriers’ customers’ positive emotions are positively related to brand images.

Customers’ perceptions of brand image could affect their perception of the quality of a specific brand by recalling past experiences, which could influence their brand attitudes (Grubb and Grathwohl, 1967). Specifically, an individual’s attitude toward specific brands could evoke the halo-effect, facilitating positive attitudes toward them. Previous studies provide the support to the positive relationship between brand image and attitudes. For instance, Richard and Allaway (1993), while exploring the relationship between service quality and selective behaviors, provide the support that customers who have a positive brand image toward a specific brand are more likely to form positive brand attitudes. Similarly, Nguyen and Leblanc (2001) also showed that an organization’s brand image affects one’s attitudes toward a specific organization. Also, Chung et al. (2009) demonstrated that positive brand images increase positive attitudes because it affects customers’ decisions to purchase products or services. Hence, customers who have positive images toward related low-cost carriers are likely to amplify their positive experiences of compassion (i.e., receiving compassion from low-cost carriers and their employees) leading to the formation of positive brand attitudes toward related low-cost carriers.

*H4*: Low-cost carriers’ customers’ brand images are positively related to brand attitudes.

Building up the previous hypotheses, we propose that positive emotions and brand image sequentially mediate the relationship between compassion and brand attitude. The affective events theory (Weiss and Cropanzano, 1996) suggests that the compassion customers experience from low-cost carriers would be recognized as an event entailing temporal, mental, and material care services, forming a positive emotion. Per the broaden-and-build model (Fredrickson, 1998, 2001), positive emotion that low-cost carriers’ customers developed through compassion experience would lead them to build a favorable brand image and attitudes of the low-cost carrier. In other words, positive emotions arouse individual attention and improve the brand image of low-cost carriers, leading customers to form an overall positive attitude toward them. Therefore, we hypothesize the following:

*H5*: Low-cost carriers’ customers’ positive emotions and brand image sequentially mediate the positive relationship between compassion and brand attitudes.

## Materials and Methods

### Study Participants and Procedure

Data were collected by inviting 460 Koreans who use low-cost carriers for an online survey. Specifically, we used the simple random sampling method for recruiting the study participants (i.e., those who have experiences of using low-cost carriers in their 20s or older), and they completed the online surveys during the first 2 weeks in December, 2020. Participants were asked to participate voluntarily and informed of their confidentiality. In addition, they provided their written informed consent online to participate in this study. While completing the surveys, participants were asked to provide their responses for the survey items based on their experiences of using low-cost carriers in general. Upon the competing of the surveys, they received mobile gift cards. Among them, 445 responded to the survey, resulting in a response rate of 96.7%. After excluding 22 surveys (e.g., responses with missing records or a central tendency bias), the study finally considered 423 surveys for the data analysis.

The sample consisted of 218 men (48.7%) and 230 women (51.3%). Eighty-four respondents were in their 20s (18.8%), 90 in their 30s (20.1%), 91 in their 40s (20.3%), 91 in their 50s (20.3%), and 92 in their 60s (20.5%). Sixty-three respondents had an education level lower than high school (14.1%), 72 were two-year community college graduates (16.1%), 280 were four-year college graduates (62.5%), and 33 were postgraduates (7.4%).

### Measures

#### Compassion

Three items developed by Lilius et al. (2008) to measure compassion were adapted to this study. They are “an experience of compassion from flight attendants on board (when using a low-cost airline, I often get temporal, material, and mental help from flight attendants when I am in trouble.);” “an experience of compassion while booking a flight (when using a low-cost airline, I often get temporal, material, and mental help from airlines when I’m in trouble.);” and “an experience of compassion from airline ground crew (when using a low-cost airline, I often get temporal, material, and mental help from the ground crew.),” all rated on a 5-point Likert scale from “strongly disagree” to “strongly agree.” The correlation among the items was acceptable (Cronbach’s *α* = 0.858).

#### Positive Emotion

This study defines positive emotion as a psychological and emotional state of a positive mood based on the customer’s experiences of a product or service (Tsaur et al., 2015). Four items used by Lilius et al. (2008) to measure the construct of positive emotions were adapted to this study (e.g., “I am proud of myself when using a low-cost airline” and “I feel joy when using a low-cost airline”). The items were rated on a 5-point Likert scale from “strongly disagree” to “strongly agree.” The correlation among the items was strong (Cronbach’s *α* = 0.857), confirming a reliable construct of positive emotions.

#### Brand Image

Drawing on Keller (1993, 1998), this study defines brand image as the perceptions about a brand as reflected by the brand associations held in consumer memory. Eight items were adapted from Driesener and Romaniuk’s (2006) study to measure the construct of brand image (e.g., “I think I have some confidence in low-cost carriers” and “I think the customer service of low-cost carriers is good”). The items were rated on a 5-point Likert scale from “strongly disagree” to “strongly agree.” The correlation among the items was strong (Cronbach’s *α* = 0.893), confirming the reliability of the construct of brand image.

#### Brand Attitude

This study adopted four items from Batra and Ray (1986), Mitchell (1986), and Keller and Aaker (1992) (e.g., “I got a good feeling about the low-cost carriers brand I used” and “Compared to other airlines, I like the low-cost carriers brand I used”) to measure brand attitude. All four items were rated on a 5-point Likert scale from “strongly disagree” to “strongly agree.” The reliability was strong enough to measure the construct of brand attitude (Cronbach’s *α* = 0.872).

We collected the demographic variables (i.e., gender, age, and education) of our study participants for controlling their potential effects on our hypothesized relationships. However, none of the demographic variables were not significantly correlated with any of our study variables. Thus, following the recommendations for the use of control variables (Becker, 2005; Spector and Brannick, 2011; Becker et al., 2016). Even when including the demographic variables in our analyses, the results showed a consistent pattern with those of our main analysis (results are available upon request to the corresponding author).

## Results

### Confirmatory Factor Analysis

Confirmatory factor analysis results are presented in Table 1. This study used the average variance extracted (AVE) and Cronbach’s Alpha (*α*) to test each latent variable’s discriminant validity and reliability, respectively. Cronbach’s *α* and AVEs were greater than the conventionally recommended thresholds of 0.7 and 0.6, respectively, for all latent variables. The results of the confirmatory factor analysis are as follows: *χ*^2^ (142) = 313.779, *p* < 0.001, CFI = 0.964, TLI = 0.956, GFI = 0.915, IFI = 0.964, RMSEA = 0.054, and RMR = 0.021, satisfying the traditional criteria (Fornell and Larcker, 1981).

**Table 1:** Confirmatory factor analysis results.

Construct	Items	*λ*	SE	CR	Cronbach’s *α*	CR
Compassion	Com1	0.826	–	–	0.858	0.811
	Com2	0.862	0.057	18.941		
	Com3	0.769	0.051	16.965		
Positive emotion	PE1	0.818	–	–	0.857	0.837
	PE2	0.823	0.061	18.862		
	PE3	0.786	0.057	17.775		
	PE4	0.68	0.052	14.835		
Brand image	BI1	0.786	–	–	0.893	0.869
	BI2	0.754	0.056	16.503		
	BI3	0.637	0.06	13.600		
	BI4	0.767	0.057	17.002		
	BI5	0.756	0.059	16.558		
	BI6	0.76	0.055	16.811		
	BI7	0.592	0.059	12.466		
	BI8	0.665	0.051	14.355		
Brand attitude	BA1	0.824	–	–	0.872	0.850
	BA2	0.779	0.06	17.871		
	BA3	0.773	0.055	17.664		
	BA4	0.808	0.054	18.759		

### Common Method Bias

We collected the data from the same sources using a cross-sectional research design, raising potential issues of common method bias. Hence, per Podsakoff et al. (2003) suggestion, we used the latent variable approach. That is, we attempted to identify if common method exists or not by comparing the changes of df and *χ*^2^ between before and after controlling the latent variables (Williams and Anderson, 1994; Podsakoff et al., 2003).

Latent variable analyses results are presented in Table 2. The model fit indices for the measurement model (i.e., before controlling the latent variables) were *χ*^2^ (142) = 313.779, *p* < 0.001, CFI = 0.964, TLI = 0.956, GFI = 0.915, IFI = 0.964, RMSEA = 0.054, and RMR = 0.021. The model fit indices for the controlled model (i.e., after controlling the latent variables) were *χ*^2^ (123) = 174.348, *p* < 0.01, CFI = 0.989, TLI = 0.985, GFI = 0.958, IFI = 0.989, RMSEA = 0.031, and RMR = 0.013. The *χ*^2^ difference (df = 19) was not statically significant, indicating that our study does not suffer from serious common method bias.

**Table 2:** Analysis of common method bias.

	*χ*^2^	df	*p*	*χ*^2^/df	RMSEA	CFI	GFI	TLI
Measurement model	313.779	142	<0.001	2.209	0.054	0.964	0.915	0.956
Controlled model	174.348	123	<0.01	1.417				
Stepwie *χ*^2^ analysis	⊿*χ*^2^	⊿df		Accepted model				
M.M-C.M	139.431	19	>0.05	Measurement model				

### Hypotheses Testing

Table 3 presents the means, standard deviations, and correlations. The variance inflation factor (VIF) of our independent variable, mediators, and the dependent variable was used as an index to test the potential multicollinearity. The VIF values ranged from 1.451 to 1.930. Thus, multicollinearity among our study variables was not an issue. Consistent with our predictions, compassion was positively related to the positive emotion (*r* = 0.532, *p* < 0.01). Additionally, positive emotion was positively related to brand image (*r* = 0.682, *p* < 0.01) and brand image to brand attitude (*r* = 0.764, *p* < 0.01).

**Table 3:** Descriptive statistics.

S. no.	Variable	Mean	SD	1	2	3	4
1.	Compassion	3.952	0.840	*0.837*			
2.	Positive emotion	3.163	0.662	0.532^**^	*0.850*		
3.	Brand image	3.347	0.542	0.472^**^	0.682^**^	*0.872*	
4.	Brand attitude	3.309	0.483	0.434^**^	0.633^**^	0.764^**^	*0.886*

** *p <* 0.01, the number in the diagonal is the square root of the AVE.

**Figure 2:**
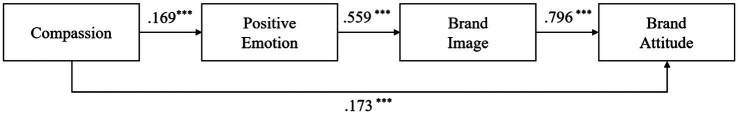
Results for hypothesized model. ^***^p < 0.001.

We used path analysis to test Hypotheses 1 through 4. Table 4 and Figure 2 summarize the path analysis results. Hypothesis 1 on the effect of compassion experienced by low-cost carriers’ customers on brand attitude was supported (*β* = 0.173, SE = 0.042, CR = 4.163, *p* < 0.001). Hypothesis 2 on the effect of compassion experienced by customers on positive emotions was also supported (*β* = 0.169, SE = 0.013, CR = 12.923, *p* < 0.001). Hypothesis 3 on the effect of positive emotion felt by customers on brand image was also supported, that is, the path coefficient from positive emotion to brand image was significant (*β* = 0.559, SE = 0.029, CR = 19.146, *p* < 0.001). Finally, Hypothesis 4 on brand image’s effect on brand attitude was also supported (*β* = 0.796, SE = 0.191, CR = 14.677, *p* < 0.001).

**Table 4:** Path analysis results.

Hypothesis	Path	*b*	SE	CR	*p*
H1	Compassion → brand attitude	0.173	0.042	4.163	*p* < 0.001
H2	Compassion → positive emotion	0.169	0.013	12.923	*p* < 0.001
H3	Positive emotion → brand image	0.559	0.029	19.146	*p* < 0.001
H4	Brand Image → brand attitude	0.796	0.191	14.677	*p* < 0.001

Bootstrapping procedures were used to test Hypothesis 5, the double mediation hypothesis. Specifically, the PROCESS by Preacher and Hayes (2004, 2008) was used. Table 5 depicts the indirect effect of compassion on brand attitudes *via* our mediators. It shows that the indirect effect of compassion on brand attitudes *via* positive emotions and brand image is 0.232 (SE = 0.032, CI = 0.173, 0.297). As the confidence interval does not include zero, our data support Hypothesis 5.

**Table 5:** Indirect effects for the double mediation effects (positive emotion and brand image).

Path	Effect	LLCI 95%	ULCI 95%	Boot SE
Total indirect effect	0.464	0.372	0.560	0.049
Compassion → PE → BA	0.121	0.053	0.199	0.037
Compassion → BI → BA	0.112	0.046	0.183	0.035
Compassion → PE → BI → BA	0.232	0.173	0.297	0.032

PE, positive emotion; BA, brand attitude; BI, brand image.

## Discussion

### Theoretical Implications

Based on the empirical results, this study has the following theoretical implications. First, the current study is one among the limited studies that examine the effects of customers’ experiences of compassion. Studies examining the causal relationship between compassion and the resulting positive outcome variables have been scarce in the field of business administration (Lilius et al., 2008; Moon et al., 2016; Ko and Choi, 2019) and focused on organizational members as subjects experiencing compassion. For example, studies on compassion in the existing business context have been centered on insiders by examining compassion experienced by organizational members, their positive emotion (Lilius et al., 2008), positive psychological capital (Ko and Choi, 2019), and positive work-related identity (Hur et al., 2016). Previous studies exploring the roles of compassion experiences from customers’ perspectives are somewhat limited. Moreover, a majority of them were conducted in healthcare settings (e.g., Austin, 2011; Kim and Choi, 2019) or regarding customers’ complaints toward the services or products they used (e.g., Stephens and Gwinner, 1998; Hwang and Mattila, 2020). This study contributes to the compassion-related literature in the existing business context. It presents the empirical examination of compassion experienced by outsiders (i.e., customers who use low-cost carriers) and its effect on brand attitude.

Furthermore, this study enhances our understanding of customers’ compassion by investigating the mechanisms for the effect of compassion on brand attitude among customers, namely members outside the organization. Specifically, although previous studies in the aviation industry explored the roles of in-flight service quality in explaining passengers’ brand attitudes and images (e.g., Han et al., 2019), they merely measured passengers’ overall perceptions of service quality rather than looking much deeper into their psychological experiences (i.e., compassion).

Finally, this study presents a new topic called compassion to the existing literature on customer satisfaction and loyalty in the airline industry as this study was conducted with low-cost carriers’ customers.

### Practical Implications

The practical implications of this study are as follows. Low-cost carriers have been less competitive than full-service carriers due to low-quality services and high delay rates. Therefore, many low-cost carriers actively implement strategies from a marketing perspective to improve their brand image to have a strategic, competitive advantage over full-service carriers. The results suggest that a strategy allowing customers to experience compassion by providing empathetic airline services should be in place to build a favorable brand attitude and image of low-cost carriers. Specifically, an empathy education program at the workplace can strengthen low-cost carriers’ competitiveness. An empathy training program offered to the ground crew and flight attendants can potentially establish an empathetic service culture for customers. If the perception that low-cost carriers provide sincere and empathetic services is instilled in customers using low-cost carriers, they will feel empathy and positive emotion toward the airlines’ services, resulting in the behavioral intention to continue to use low-cost airlines in the future.

### Limitations and Future Research Directions

This study is not without limitations. First, this study used cross-sectional data, limiting the claim of causality between latent variables. In addition, this study collected the data from single sources which raises a potential common method bias issue. Although we conducted the latent variable analysis following Podsakoff et al. (2003), we acknowledge that we cannot completely rule of the possibility of common method in our research model. As such, given that compassion is experienced by low-cost carriers’ customers, and positive emotion and brand image are formed and maintained over a considerable period, future research will benefit by adopting the longitudinal research design to warrant the much clearer causal relationships and their changes over time (Sierra et al., 2010). Furthermore, the future research could also explore the actual behaviors of low-cost carriers’ customers, such as returning to the low-cost carriers services, as focal outcomes extending our findings.

Second, this is the first study to empirically investigate the causal relationships between compassion and its outcome variables among low-cost carriers’ customers. Consequently, we adapted an existing scale to measure compassion in the context of low-cost carriers. Thus, it is necessary to validate the findings of this study through various methods. Although empirical studies on compassion have progressed steadily (Lilius et al., 2008; Hur et al., 2016; Moon et al., 2016; Ko and Choi, 2019), future studies need to employ a mixed-methods approach to secure the validity of the scale to measure compassion. Third, this study modeled compassion, positive emotion, and brand image as the antecedents of brand attitude. However, future studies need to consider the possibility that compassion can affect the perceived quality of the company’s services and brand preference. Therefore, studies need to go beyond the mechanisms empirically investigated in this study and examine new mechanisms. Finally, the generalizability of this study’s findings is limited because the sample includes only customers residing in South Korea using low-cost carriers. Therefore, future research needs to include samples from various countries for comparison and to arrive at more generalizable findings. Furthermore, a comparative study of compassion between customers from multiple countries using full-service and low-cost carriers should be conducted.

## Conclusion

This study theoretically and empirically investigated the effect of compassion experienced by low-cost carriers’ customers on brand attitude through positive emotion and brand image. The empirical results confirmed that customers’ compassion, experienced during the overall process of using low-cost carriers’ services, helps them form a positive brand attitude toward the carriers. Particularly, this study found that customers’ positive emotions and brand image play an essential role in the mechanism through how compassion leads to the formation of positive brand attitudes. Customers who perceive that low-cost carriers are actively engaged in empathetic air service are more likely to form positive emotions, encouraging them to have a more favorable brand image, consequently building a positive brand attitude. Given the evolving competition in the low-cost carrier industry, the results from this study contribute to the literature on the airline industry by suggesting fruitful ways to explore the role of compassion and provide practical insights for low-cost carriers to gain a competitive edge while maintaining price competitiveness.

## Data Availability Statement

The raw data supporting the conclusions of this article will be made available by the authors, without undue reservation.

## Ethics Statement

Ethical review and approval was not required for the study on human participants in accordance with the local legislation and institutional requirements. The patients/participants provided their written informed consent to participate in this study.

## Author Contributions

S-HK and JK conceived the presented model and developed the theory. S-HK collected the data. S-HK and YC analyzed the data and wrote the original manuscript with input from all authors. JK aided in interpreting the results and worked on the practical discussion. All authors contributed to manuscript revision, read, and approved the submitted version.

### Conflict of Interest

The authors declare that the research was conducted in the absence of any commercial or financial relationships that could be construed as a potential conflict of interest.

## References

[ref1] Alexandris K. Douka S. Papadopoulos P. Kaltsatou A. (2008). Testing the role of service quality on the development of brand associations and brand loyalty. Manag. Serv. Qual. An Intern. J. 24, 112–127. 10.1108/09604520810871865

[ref2] Austin W. J. (2011). The incommensurability of nursing as a practice and the customer service model: an evolutionary threat to the discipline. Nurs. Philos. 12, 158–166. 10.1111/j.1466-769x.2011.00492.x, PMID: 21668615

[ref3] Baker D. Mc A. (2013). Service quality and customer satisfaction in the airline industry: a comparison between legacy airlines and low-cost airlines. Am. J. Tourism Res. 2, 67–77. 10.11634/216837861302317

[ref4] Baker D. Mc A. (2014). Low-cost airlines management model and customer satisfaction. Int. J. Econ. Comm. Manag. 2, 1–17.

[ref5] Batra R. Ray M. L. (1986). Affective responses mediating acceptance of advertising. J. Consum. Res. 13, 234–249. 10.1086/209063

[ref6] Baumann C. Burton S. Elliott G. Kehr H. (2007). Prediction of attitude and behavioural intentions in retail banking. Int. J. Bank Mark. 25, 102–116. 10.1108/02652320710728438

[ref7] Becker T. E. (2005). Potential problems in the statistical control of variables in organizational research: a qualitative analysis with recommendations. Organ. Res. Methods 8, 274–289. 10.1177/1094428105278021

[ref8] Becker T. E. Atinc G. Breaugh J. A. Carlson K. D. Edwards J. R. Spector P. E. (2016). Statistical control in correlational studies: 10 essential recommendations for organizational researchers. J. Organ. Behav. 37, 157–167. 10.1002/job.2053

[ref9] Bejou D. Palmer A. (1998). Service failure and loyalty: an exploratory empirical study of airline customers. J. Serv. Mark. 12, 7–22. 10.1108/08876049810202339

[ref10] Bor R. (2007). Psychological factors in airline passenger and crew behaviour: a clinical overview. Travel Med. Infect. Dis. 5, 207–216. 10.1016/j.tmaid.2007.03.003, PMID: 17574141

[ref11] Carlson J. O’Cass A. (2010). Exploring the relationships between e-service quality, satisfaction, attitudes and behaviours in content-driven e-service web sites. J. Serv. Mark. 24, 112–127. 10.1108/08876041011031091

[ref12] Childs M. Kim S. (2019). Exploring conspicuous compassion as a brand management strategy. J. Prod. Brand. Manag. 28, 540–554. 10.1108/JPBM-05-2018-1882

[ref13] Chung J. E. Pysarchik D. T. Hwang S.-J. (2009). Effects of country-of-manufacture and brand image on Korean consumers’ purchase intention. J. Glob. Mark. 22, 21–41. 10.1080/08911760802511352

[ref14] Curras-Perez R. Sanchez-Garcia I. (2016). Antecedents and consequences of consumer commitment in traditional and low-cost airlines. J. Travel Tourism. Mark. 33, 899–911. 10.1080/10548408.2015.1075458

[ref15] Dick A. S. Basu K. (1994). Customer loyalty: toward an integrated conceptual framework. J. Acad. Market Sci. 22, 99–113. 10.1177/0092070394222001

[ref16] Dobruszkes F. (2006). An analysis of European low-cost airlines and their networks. J. Transp. Geogr. 14, 249–264. 10.1016/j.jtrangeo.2005.08.005

[ref17] Driesener C. Romaniuk J. (2006). Comparing methods of brand image measurement. Int. J. Mark. Res. 48, 681–698. 10.1177/147078530604800605

[ref18] Dutton J. E. (2003). Energize Your Workplace: How to Build and Sustain High-Quality Connections at Work. San Francisco: Jossey-Bass.

[ref19] Dutton J. E. Roberts L. M. Bednar J. (2010). Pathways for positive identity construction at work: four types of positive identity and the building of social resources. Acad. Manag. J. 35, 265–293. 10.5465/amr.35.2.zok265

[ref20] Dutton J. E. Workman K. M. Hardin A. E. (2014). Compassion at work. Annu. Rev. Organ. Psych. Organ. Behav. 1, 277–304. 10.1146/annurev-orgpsych-031413-091221

[ref21] Ephron E. (2006). Media Planning: From Recency to Engagement. India: ICFAI University Press.

[ref22] Faircloth J. B. Capella L. M. Alford B. L. (2001). The effect of brand attitude and brand image on brand equity. J. Mark. Theory Pract. 9, 61–75. 10.1080/10696679.2001.11501897

[ref23] Fishbein M. (1963). An investigation of the relationships between beliefs about an object and the attitude toward that object. Hum. Relat. 16, 233–239. 10.1177/001872676301600302

[ref24] Folkman S. Moskowitz J. T. (2000). Positive affect and the other side of coping. Am. Psychol. 55, 647–654. 10.1037//0003-066x.55.6.647, PMID: 10892207

[ref25] Fornell C. Larcker D. F. (1981). Evaluating structural equation models with unobservable variables and measurement error. J. Mar. Res. 18, 39–50. 10.1177/002224378101800104

[ref26] Fredrickson B. L. (1998). What good are positive emotions? Rev. Gen. Psychol. 2, 300–319. 10.1037/1089-2680.2.3.300, PMID: 21850154 PMC3156001

[ref27] Fredrickson B. L. (2001). The role of positive emotions in positive psychology, the broaden-and-build theory of positive emotions. Am. Psychol. 56, 218–226. 10.1037//0003-066x.56.3.218, PMID: 11315248 PMC3122271

[ref28] Frost P. J. (2003). Toxic Emotions at Work: How Compassionate Managers Handle Pain and Conflict. Boston, MA: Harvard Business School Press.

[ref29] Gracia E. Bakker A. B. Grau R. M. (2011). Positive emotions: the connection between customer quality evaluations and loyalty. Cornell Hosp. Q. 52, 458–465. 10.1177/1938965510395379

[ref30] Grubb E. L. Grathwohl H. L. (1967). Consumer self-concept, symbolism and market behavior: a theoretical approach. J. Mark. 31, 22–27. 10.1177/002224296703100405

[ref31] Hallowell E. M. (1999). The human moment at work. Harv. Bus. Rev. 77, 58–66. PMID: 10345392

[ref32] Han H. Hyun S. Kim W. (2014). In-flight service performance and passenger loyalty: a cross-national (China/Korea) study of travelers using low-cost carriers. J. Travel Tourism Market. 31, 1–21. 10.1080/10548408.2014.883954

[ref33] Han H. Yu J. Chua B. L. Lee S. Kim W. (2019). Impact of core-product and service-encounter quality, attitude, image, trust and love on repurchase: full-service vs. low-cost carriers in South Korea. Int. J. Contemp. Hosp. Manag. 31, 1588–1608. 10.1108/IJCHM-05-2018-0376

[ref34] He Y. Chen Q. Alden D. L. (2016). Time will tell: managing post-purchase changes in brand attitude. J. Acad. Mark. Sci. 44, 791–805. 10.1007/s11747-015-0444-7

[ref35] Heller J. (2003). “Psychological and psychiatric difficulties among airline passengers,” in Passenger Behaviour. ed. Bor R. (Hampshire: Ashgate Publishing), 60–65.

[ref36] Hemsley-Brown J. Alnawas I. (2016). Service quality and brand loyalty: the mediation effect of brand passion, brand affection and self-brand connection. Int. J. Contemp. Hosp. Manag. 28, 2771–2794. 10.1108/IJCHM-09-2015-0466

[ref37] Hur W. M. Moon T. Rhee S. Y. (2016). Exploring the relationships between compassion at work, the evaluative perspective of positive work-related identity, service employee creativity, and job performance. J. Serv. Mark. 30, 103–114. 10.1108/JSM-05-2014-0180

[ref38] Hwang Y. Mattila A. S. (2020). The impact of customer compassion on face-to-face and online complaints. J. Hosp. Mark. Manag. 29, 848–868. 10.1080/19368623.2020.1711546

[ref39] Kahn W. A. (1993). Caring for the caregivers: patterns of organizational caregiving. Adm. Sci. Q. 38, 539–563. 10.2307/2393336

[ref40] Kahn W. A. (1998). “Relational systems at work,” in Research in Organizational Behavior. eds. Staw B. M. Cummings L. L. (New York: Elsevier Science), 39–76.

[ref41] Keller K. L. (1993). Conceptualizing, measuring, and managing customer-based brand equity. J. Mark. 57, 1–22. 10.1177/002224299305700101

[ref42] Keller K. L. (1998). Strategic Brand Management: Building, Measuring, and Managing Brand Equity. Englewood Cliffs, NJ: Prentice Hall.

[ref43] Keller K. L. Aaker D. A. (1992). The effects of sequential introduction of brand extensions. J. Mar. Res. 29, 35–50. 10.1177/002224379202900104

[ref44] Khan N. T. Jung G. Kim J. Kim Y. B. (2019). Evolving competition between low-cost carriers and full-service carriers: the case of South Korea. J. Transp. Geogr. 74, 1–9. 10.1016/j.jtrangeo.2018.10.017, PMID: 32288378 PMC7125652

[ref45] Kim Y. H. Choi M. Y. (2019). Effects of compassion competence and organizational commitment on customer orientation in hospital nurses. J. Kor. Clin. Nurs. Res. 25, 133–141. 10.22650/JKCNR.2019.25.2.133

[ref46] Ko S. H. Choi Y. (2019). Compassion and job performance: dual-paths through positive work-related identity, collective self esteem, and positive psychological capital. Sustain. For. 11:6766. 10.3390/su11236766

[ref47] Koklic M. K. Kukar-Kinney M. Vegelj S. (2017). An investigation of customer satisfaction with low-cost and full-service airline companies. J. Bus. Res. 80, 188–196. 10.1016/j.jbusres.2017.05.015

[ref48] Lilius J. M. Worline M. C. Maitlis S. Kanov J. Dutton J. E. Frost P. (2008). The contours and consequences of compassion at work. J. Organ. Behav. 29, 193–218. 10.1002/job.508

[ref49] Lutz R. J. (1975). Changing brand attitudes through modification of cognitive structure. J. Consum. Res. 1, 49–59. 10.1086/208607

[ref50] Mehrabian A. Russell J. A. (1974). An Approach to Environmental Psychology. Cambridge, MA: MIT Press.

[ref51] Meyer F. Huber F. Huber S. (2019). The suffering company: consumer compassion towards companies exposed to negative events. Psychol. Mark. 36, 321–341. 10.1002/mar.21181

[ref52] Mitchell A. A. (1986). The effect of verbal and visual components of advertisements on brand attitudes and attitude toward the advertisement. J. Consum. Res. 13, 12–24. 10.1086/209044

[ref53] Moon T. W. Hur W. M. Ko S. H. Kim J. W. Yoo D. K. (2016). Positive work-related identity as a mediator of the relationship between compassion at work and employee outcomes. Hum. Fact. Ergon. Man. 26, 84–94. 10.1002/hfm.20615

[ref54] Nguyen N. Leblanc G. (2001). Corporate image and corporate reputation in customers’ retention decisions in services. J. Retail. Consum. Serv. 8, 227–236. 10.1016/S0969-6989(00)00029-1

[ref55] Oliver R. L. (1980). A cognitive model of the antecedents and consequences of satisfaction decisions. J. Mar. Res. 17, 460–469. 10.1177/002224378001700405

[ref56] Pan J. Y. Truong D. (2018). Passengers’ intentions to use low-cost carriers: an extended theory of planned behavior model. J. Air Transp. Manag. 69, 38–48. 10.1016/j.jairtraman.2018.01.006

[ref57] Pearson J. Pitfield D. Ryley T. (2015). Intangible resources of competitive advantage: analysis of 49 Asian airlines across three business models. J. Air Transp. Manag. 47, 179–189. 10.1016/j.jairtraman.2015.06.002

[ref58] Podsakoff P. M. MacKenzie S. B. Lee J. Y. Podsakoff N. P. (2003). Common method biases in behavioral research: a critical review of the literature and recommended remedies. J. Appl. Psychol. 88, 879–903. 10.1037/0021-9010.88.5.879, PMID: 14516251

[ref59] Preacher K. J. Hayes A. F. (2004). SPSS and SAS procedures for estimating indirect effects in simple mediation models. Behav. Res. Meth. Ins. C. 36, 717–731. 10.3758/bf03206553 15641418

[ref60] Preacher K. J. Hayes A. F. (2008). Asymptotic and resampling strategies for assessing and comparing indirect effects in multiple mediator models. Behav. Res. Methods 40, 879–891. 10.3758/brm.40.3.879, PMID: 18697684

[ref61] Richard M. D. Allaway A. W. (1993). Service quality attributes and choice behavior. J. Serv. Mark. 7, 59–68. 10.1108/08876049310026105

[ref62] Sallam M. A. (2014). The effects of brand image and brand identification on brand love and purchase decision making: the role of WOM. Int. Bus. Res. 7, 187–193. 10.5539/ibr.v7n10p187

[ref63] Sierra J. J. Heiser R. S. Williams J. D. Taute H. A. (2010). Consumer racial profiling in retail environments: a longitudinal analysis of the impact on brand image. J. Brand Manag. 18, 79–96. 10.1057/bm.2010.24

[ref64] Spector P. E. Brannick M. T. (2011). Methodological urban legends: the misuse of statistical control variables. Organ. Res. Methods 14, 287–305. 10.1177/1094428110369842

[ref65] Stephens N. Gwinner K. P. (1998). Why don’t some people complain? A cognitive-emotive process model of consumer complaint behavior. J. Acad. Mark. Sci. 26, 172–189. 10.1177/0092070398263001

[ref66] Tsaur S. H. Luoh H. F. Syue S. S. (2015). Positive emotions and behavioral intentions of customers in full-service restaurants: does aesthetic labor matter? Int. J. Hosp. Manag. 51, 115–126. 10.1016/j.ijhm.2015.08.015

[ref67] Weiss H. M. Cropanzano R. (1996). “Affective events theory: a theoretical discussion of the structure, causes, and con sequences of affective experiences at work,” in Research in Organizational Behavior. eds. Staw B. M. Cummings L. L. (New York: Elsevier Science), 1–74.

[ref68] Williams L. J. Anderson S. E. (1994). An alternative approach to method effects by using latent-variable models: applications in organizational behavior research. J. Appl. Psychol. 79, 323–331. 10.1037/0021-9010.79.3.323

[ref69] Wirtz J. Mattila A. S. Tan R. L. P. (2000). The moderating role of target-arousal on the impact of affect on satisfaction—an examination in the context of service experiences. J. Retail. 76, 347–365. 10.1016/S0022-4359(00)00031-2

[ref70] Wongleedee K. (2017). Customer satisfaction in the airlines industry: comparison between low-cost and full service airlines. Актуальні Проблеми Економіки 1, 218–222.

[ref71] Wuthnow R. (2012). Acts of Compassion: Caring for Others and Helping Ourselves. Princeton: Princeton University Press.

